# Biologic Augmentation for Meniscus Repair: A Narrative Review

**DOI:** 10.3390/bioengineering13010101

**Published:** 2026-01-15

**Authors:** Tsung-Lin Lee, Scott Rodeo

**Affiliations:** 1 Department of Orthopaedic Surgery, Hospital for Special Surgery, New York, NY 10021, USA; leets@hss.edu; 2 Department of Orthopaedic Surgery, Fu Jen Catholic University Hospital, New Taipei City 24352, Taiwan

**Keywords:** meniscus healing, biologic augmentation, synovial abrasion, platelet-rich plasma (PRP), biologic target, cell therapy, collagen scaffold

## Abstract

Meniscal preservation is increasingly recognized as a critical determinant of long-term knee joint health, yet successful repair remains challenging due to the meniscus’s limited intrinsic healing capacity. The adult meniscus is characterized by restricted vascularity, low cellularity, a dense extracellular matrix, complex biomechanical loading, and a hostile post-injury intra-articular inflammatory environment—factors that collectively impair meniscus healing, particularly in the avascular zones. Over the past several decades, a wide range of biologic augmentation strategies have been explored to overcome these barriers, including synovial abrasion, fibrin clot implantation, marrow stimulation, platelet-derived biologics, cell-based therapies, scaffold coverage, and emerging biologic and biophysical interventions. This review summarizes the biological basis of meniscal healing, critically evaluates current and emerging biologic augmentation techniques, and integrates these approaches within a unified framework of vascular, cellular, matrix, biomechanical, and immunologic targets. Understanding and modulating the cellular and molecular mechanisms governing meniscal degeneration and repair may enable the development of more effective, mechanism-driven strategies to improve healing outcomes and reduce the risk of post-traumatic osteoarthritis.

## 1. Introduction

The meniscus plays a fundamental role in the prevention or delay of degenerative changes through load transmission across tibio-femoral joint, joint lubrication, and enhancing joint stability. The meniscus is composed primarily of water (approximately 72% of total tissue mass), with the remaining solid phase consisting of a dense extracellular matrix (ECM) dominated by type I collagen (22% by wet weight), proteoglycans (1% by wet weight), and sparsely distributed fibrochondrocytes (meniscal cells), which are responsible for ECM synthesis [1,2].

The biomechanical function of the meniscus is largely determined by its microscopic ECM organization. Circumferentially oriented type I collagen bundles, reinforced by radial tie fibers, provide tensile strength and facilitate the effective transmission of hoop stresses arising from axial loading. Proteoglycans, although present at lower concentrations than in articular cartilage, attract water and contribute to compressive stiffness, viscoelastic behavior, and load transmission. Consequently, loss of meniscal tissue—whether from traumatic injury or surgical resection—has been consistently associated with increased contact stresses, accelerated cartilage degeneration, and the development of post-traumatic osteoarthritis. These observations have driven a paradigm shift in contemporary orthopedic practice from meniscectomy toward meniscal preservation whenever feasible.

Despite this shift, meniscal repair remains limited by the tissue’s poor intrinsic healing capacity, particularly within the inner avascular regions. As a result, tear selection remains a critical determinant of repair success, with peripheral longitudinal tears demonstrating more favorable healing than radial, complex, or central avascular lesions. However, strict reliance on tear morphology alone may unnecessarily limit repair indications, particularly in younger and active patients for whom meniscal preservation is most beneficial. Supporting this concern, the Multicenter Orthopaedic Outcomes Network (MOON) Group found that patients undergoing medial meniscus repair have worse patient-reported outcomes compared to those with uninjured menisci, indicating significant room for improvement in current treatment strategies [3].

What is increasingly recognized is that meniscal healing is inherently slow and frequently imperfect, particularly in avascular regions where intrinsic repair capacity is limited. Despite advances in arthroscopic repair techniques and regenerative approaches, the clinical need to confidently heal meniscal tears—especially complex or centrally located lesions—remains unmet, with failed healing accelerating cartilage degeneration and post-traumatic osteoarthritis.

At the same time, the optimal strategy for biologically augmenting meniscal repair remains unknown due to an incomplete understanding of the underlying biology. The identity and origin of reparative cell populations, as well as the relative importance of key biological processes such as cell proliferation, angiogenesis, extracellular matrix synthesis, cell chemotaxis, and inflammatory modulation, remain poorly defined. This knowledge gap underscores the need for a mechanism-based framework to guide rational selection and development of biologic augmentation strategies. These challenges have driven increasing interest in biologic augmentation strategies designed to enhance healing. The purpose of this review is to (1) summarize the biological factors that limit meniscal healing, (2) critically evaluate established and emerging biologic augmentation strategies, (3) propose a practical, mechanism-based approach to optimize meniscal repair, and (4) outline future directions for the development of biologic therapies aimed at improving meniscal healing outcomes.

This narrative review was informed by searches of PubMed/MEDLINE and Google Scholar for English-language, preclinical and clinical studies related to meniscal healing and biologic augmentation (e.g., “meniscus repair,” “biologic augmentation,” “fibrin clot,” “marrow venting,” “PRP,” “synovial abrasion,” “cell therapy,” “scaffold”). Seminal and frequently cited foundational studies were prioritized, and additional references were identified by screening bibliographies of relevant reviews and primary studies.

## 2. Biology of Meniscus Healing

### 2.1. Limited Vascularity

**Figure 1:**
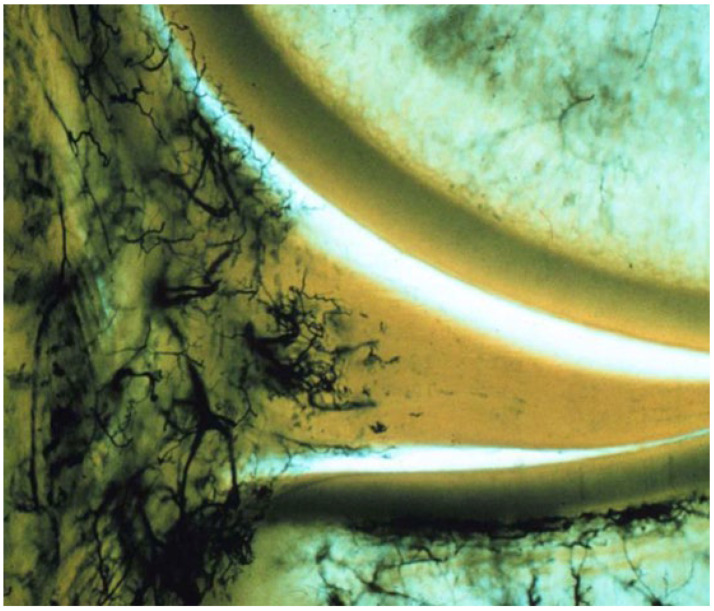
Histologic demonstration of meniscal vascularity showing blood supply confined predominantly to the peripheral one-third of the medial meniscus. Reproduced with permission from Arnoczky and Warren [7], The American Journal of Sports Medicine, 1982.

The evidence clearly demonstrates that the meniscus has limited vascularity, with blood vessels primarily confined to the outer (peripheral) region. Multiple anatomical and histological studies show that in adults, vascular penetration typically reaches only the outer 10–33% of the meniscus, while the inner two-thirds remain largely avascular [4,5,6]. For example, Crawford et al. found that in young adults, the maximal depth of vascular penetration ranged from 0% to 42% in the medial meniscus and 0% to 48% in the lateral meniscus, with the posterior horn of medial meniscus being the least vascularized [4]. Michel et al. further quantified that after adolescence, no blood vessels are found in the inner (white-white) and intermediate (red-white) zones, and almost all vessels are located only in the capsule or outermost region [5]. Representative histologic images from the classic vascular mapping study by Arnoczky and Warren illustrate that vascular penetration is largely restricted to the peripheral one-third of the meniscus [7] (Figure 1). This limited vascularity of the meniscus, especially in the inner zones, is a key reason for its poor intrinsic healing capacity after injury.

### 2.2. Hypocellular

The meniscus is relatively hypocellular, especially in its central regions, as demonstrated by histological and quantitative studies. The meniscal body consists mainly of extracellular matrix with sparse cellularity, and this low cell density is a well-established anatomical feature [1]. Quantitative histomorphometric analyses show that the vascular (peripheral) region of the meniscus has higher cellular density (e.g., ~27,000 cells/mm^3^), while the avascular (central) region is significantly less cellular (e.g., ~13,000 cells/mm^3^) [8].

Cellularity also decreases with age and in degenerative conditions, further reducing the meniscus’s intrinsic healing capacity [9,10]. The meniscus is primarily composed of fibrochondrocytes, which are distributed sparsely within a dense collagen matrix, and the tissue’s hypocellularity is most pronounced in the inner, avascular zones, as well as anterior segments [1,8,11]. This structural characteristic is a key reason for the meniscus’s limited ability to repair itself after injury.

### 2.3. Dense Extracellular Matrix

Beyond cellular and vascular constraints, the dense extracellular matrix (ECM) of the adult meniscus constitutes a physical and biophysical barrier that limits cell chemotaxis and migration. Experimental investigations have demonstrated that the tightly packed collagen architecture and high matrix density characteristic of the mature meniscal ECM mechanically restrict interstitial cell movement. For instance, Qu et al. showed that whereas the fetal meniscus—characterized by a looser and less organized ECM—allows robust cellular migration and intrinsic repair, the adult meniscal matrix markedly impedes these processes [12]. Enzymatic alteration of the adult ECM, such as treatment with hyaluronidase or collagenase, increases matrix porosity and mechanical compliance, thereby promoting cellular migration within the tissue and enhancing reparative responses [12,13,14]. However, such treatments may adversely affect the material properties of the tissue.

In a similar way, Yan et al. reported that transforming the dense and stiff adult meniscal matrix into a more fetal-like, compliant, and loosely organized microenvironment using hyaluronidase and TGF-β3 effectively alleviates biophysical barriers to cell migration, leading to improved cellular infiltration and tissue integration at the injury interface [13]. These observations are further supported by computational simulations and in vivo animal models, which consistently indicate that ECM density itself represents a principal limitation to cell chemotaxis and repair in the adult meniscus, independent of vascular supply or cell origin [12,14].

### 2.4. Challenging Biomechanical Environment

The meniscus is exposed to a demanding biomechanical environment defined by high and complex mechanical forces, which markedly compromises its healing potential, particularly within avascular regions. The meniscus is required to withstand compressive, tensile, and shear loading during normal joint function, with peak stresses occurring during activities such as pivoting, squatting, and sports. This biomechanical milieu contributes to a high incidence of traumatic and degenerative tears, especially in areas characterized by limited vascular supply and low cellularity, where intrinsic repair is limited [15,16].

Biomechanical studies further demonstrate that meniscal cells and extracellular matrix are exposed to nonuniform stress–strain distributions, fluctuating fluid pressures, and dynamic loading, all of which may compromise tissue integrity and interfere with cell migration and matrix synthesis essential for healing [13,16]. Following injury, compromised ability to distribute load leads to increased articular cartilage strain and joint instability, which may further impair meniscus healing [17].

Clinical and epidemiological evidence further underscores the close relationship between anterior cruciate ligament (ACL) injury and meniscal pathology, highlighting the role of biomechanical instability in meniscal injury and impaired healing. Acute ACL rupture (<8 weeks) is frequently associated with lateral meniscus tears, with reported incidence rates approaching 70% [18]. These injuries are largely attributed to high-energy rotational forces and pivot-shift mechanisms at the time of ligament rupture. In contrast, chronic ACL insufficiency is more commonly associated with medial meniscus tears, which develop over time as a consequence of repetitive anterior tibial translation and increased shear forces. Hagino et al. demonstrated that isolated medial meniscus tears occurred in 24.7% of chronic ACL-deficient knees compared to 10.8% in the acute group, representing more than a twofold increase [18].

Importantly, knee stability exerts a substantial influence on meniscal healing outcomes. Multiple clinical studies have shown that meniscal repair performed in a stable knee—either with an intact ACL or in conjunction with ACL reconstruction—results in significantly higher healing rates than repairs performed in ACL-deficient knees [19]. These findings emphasize that biomechanical instability not only predisposes the meniscus to injury but also creates a mechanically unfavorable environment that compromises repair and regeneration. The biomechanical evidence demonstrates that this benefit results from elimination of pathologic gapping that mechanically prevents tissue integration, while the biological environment created by ACL reconstruction further supports the healing process [20]. Consequently, restoration of joint stability represents a critical prerequisite for successful meniscal healing and provides a strong biomechanical rationale for combining mechanical stabilization with biologic augmentation strategies.

### 2.5. Intra-Articular Inflammatory Environment

Emerging biochemical evidence highlights that meniscal injury creates a profoundly catabolic intra-articular environment that may impair tissue repair and predispose the joint to post-traumatic osteoarthritis (PTOA). Liu et al. reported that patients with isolated meniscus tears exhibit dramatically elevated levels of matrix metalloproteinase (MMP) activity and prostaglandin E_2_ (PGE_2_) in synovial fluid compared with non-osteoarthritic controls, with MMP activity increased 25-fold and PGE_2_ increased 290-fold [21]. Notably, MMP activity and PGE_2_ concentrations were strongly correlated, and both were substantially higher in synovial fluid than in serum, indicating a localized intra-articular catabolic response rather than a systemic process [21].

**Figure 2:**
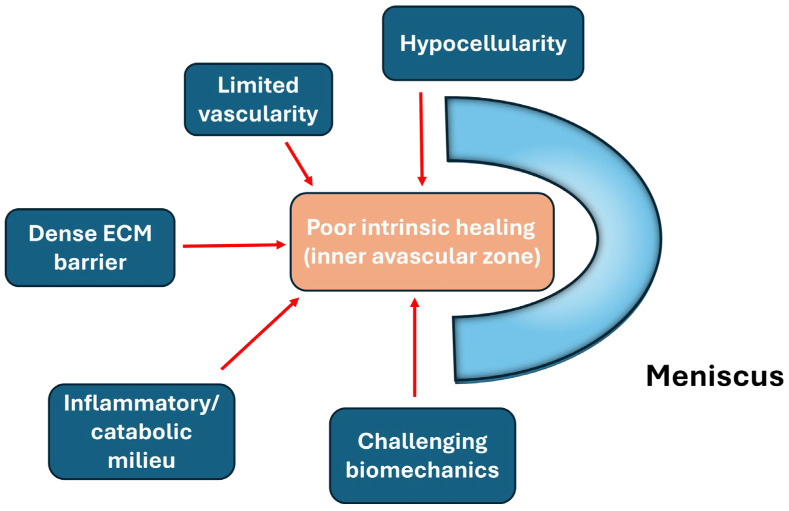
Major biological barriers limiting intrinsic meniscal healing, including limited vascularity, hypocellularity, dense extracellular matrix, biomechanical stress, and inflammatory milieu.

Hemarthrosis, the critical intra-articular pathology following meniscus injury, also plays an important role in post-injury joint inflammation. Exposure of meniscal tissue to whole blood has been shown to induce a potent catabolic response largely mediated by mononuclear leukocytes. In a porcine ex vivo meniscal explant system designed to simulate hemarthrosis and blood-derived biologics, mononuclear leukocytes were identified as the principal drivers of increased matrix metalloproteinase (MMP) activity, nitric oxide (NO) production, and accelerated sulfated glycosaminoglycan (sGAG) depletion from the meniscal extracellular matrix, indicating substantial matrix degradation and impaired tissue integrity [22]. These findings provide mechanistic support for clinical concerns that hemarthrosis following knee trauma may hinder meniscal healing, and they further underscore the need for caution when using blood-derived therapies, as leukocyte-rich preparations may inadvertently potentiate catabolic pathways within meniscal tissue [22]. Consequently, effective meniscus repair strategies must therefore not only address structural stabilization but also actively modulate the intra-articular inflammatory and catabolic milieu to prevent matrix degradation and promote tissue regeneration (Figure 2).

## 3. Biologic Augmentation Approaches

### 3.1. Synovial Abrasion

Animal and clinical studies consistently demonstrate that synovial tissue plays a critical role in meniscus healing, especially for lesions near the peripheral synovial rim, while healing is limited for tears farther from the synovium. Arnoczky and Warren’s classic dog model showed that meniscal lesions connected to peripheral synovial tissue heal by forming a fibrovascular scar, highlighting the importance of synovial-derived reparative cells and vascular ingrowth [23]. This is the earliest evidence of proliferation of vascular synovial tissue at the meniscal injury site. This foundational concept is supported by more recent experimental work, which emphasize that the synoviomeniscal junction is a key source of reparative cells and growth factors for meniscal healing [24].

Around 2000, Okuda and Ochi and their colleagues described a surgical rasping technique aimed at promoting healing of meniscal tears in the avascular zone using a rabbit model [25,26]. Their approach involved rasping not only the parameniscal synovium but also the meniscal surface itself, including the injured area, to a depth of approximately 0.5 mm. Immunohistochemical analyses demonstrated a rapid increase in transforming growth factor–β1 (TGF-β1) expression in the rasped femoral surface area within 7 days after surgery. The authors proposed that this induced cytokine–cytokine network stimulated cellular recruitment, proliferation, and collagen synthesis, thereby facilitating healing of a meniscal tear in the avascular zone [26].

However, earlier work by Nakhostine et al. in 1990 reported that meniscal tears located farther from the synovial rim did not heal effectively in a sheep model, although direct rasping of the meniscal lesion itself was not performed in that study [27]. Subsequent clinical reports have described favorable outcomes with rasping-based techniques, but these studies were limited by small sample sizes and the absence of control groups [28]. Taken together, despite heterogeneous and incomplete evidence, meniscal rasping remains a biologically plausible and potentially valuable adjunctive technique that warrants further investigation.

### 3.2. Fibrin Clot—The Earliest Approach for Biologic Augmentation of Meniscus Healing

Across animal models and clinical studies, exogenous fibrin clot consistently promotes healing of meniscal tears, particularly in the avascular regions where intrinsic repair capacity is limited. In a foundational canine model, fibrin clot placed into avascular meniscal defects induced robust repair through chemotactic recruitment of reparative cells and formation of fibrocartilaginous tissue, whereas untreated defects failed to heal [29]. Clinically, Henning and colleagues demonstrated that arthroscopic meniscal repairs augmented with fibrin clot had markedly lower failure rates in isolated tears (8% vs. 41% without clot) and improved healing even in complex tear patterns when combined with fascial sheath coverage [30,31]. Subsequent case series further showed that fibrin clot can facilitate repair of complete radial tears in the avascular lateral meniscus, with second-look arthroscopy confirming universal healing [32]. Collectively, these studies highlight fibrin clot as one of the earliest and most consistently effective biologic augmentation methods for meniscal repair, capable of extending healing potential into regions traditionally considered lacking intrinsic healing capacity.

### 3.3. Microfracture

Marrow-stimulating techniques, including microfracture of the intercondylar notch, have emerged as effective biologic augmentation strategies for meniscal repair. Freedman et al. first demonstrated that microfracture can deliver marrow elements—rich in mesenchymal stromal cells and growth factors—to the repair site, thereby enhancing healing in avascular regions of the meniscus [33]. Building upon this biologic rationale, Dean et al. showed that isolated meniscal repairs augmented with “marrow venting” achieve outcomes comparable to repairs performed in conjunction with ACL reconstruction, which naturally benefits from marrow-derived hemarthrosis [34]. Together, marrow stimulation techniques such as microfracture or marrow venting are effective, low-risk biological augmentation methods for meniscal repair, particularly in isolated tears, and can achieve outcomes comparable to repairs performed with ACL reconstruction.

### 3.4. Platelet-Rich Plasma (PRP), Platelet-Rich Fibrin (PRF), and Platelet-Rich Fibrin Matrix (PRFM)

Platelet-derived biologics have been explored as augmentation strategies for meniscal repair, with platelet-rich plasma (PRP) being the most extensively studied and platelet-rich fibrin (PRF) and platelet-rich fibrin matrix (PRFM) representing newer second-generation formulations.

High-level evidence supports a selective benefit of PRP: a randomized controlled trial demonstrated improved structural healing with leukocyte-rich PRP in unstable vertical tears [35], and several comparative studies and systematic reviews report reduced failure rates for isolated meniscal repairs augmented with PRP [36,37,38], although PRP provides no additional benefit when repairs are performed with concomitant ACL reconstruction [36]. However, a large database study performed at Mayo Clinic reports the opposite pattern: Dancy et al. found no reduction in revision surgery rates for isolated meniscal repairs augmented with PRP or bone marrow aspirate concentrate (BMAC), but identified a modest reduction in revisions when biologics were used in the setting of concomitant ACL reconstruction—although the absolute number of events was small [39].

In contrast, evidence for PRF remains primarily preclinical. Animal studies demonstrate that PRF enhances meniscus cell proliferation, migration, and extracellular matrix synthesis and serves as a bioactive scaffold with sustained growth factor release [40,41]. However, systematic reviews reveal a lack of clinical trials evaluating PRF for meniscal repair, and contemporary practice surveys show that PRF is used infrequently relative to mechanical or marrow-stimulation augmentation techniques [42,43]. Platelet-rich fibrin matrix (PRFM), a leukocyte-poor fibrin matrix produced via calcium-induced polymerization of anticoagulated blood, offers a denser and more uniform fibrin architecture with controlled growth factor release. However, recent animal data do not demonstrate clear benefit: a rabbit model comparing PRP, PRFM, and meniscal repair alone found no significant differences in defect filling, collagen formation, or cell morphology among MR (Meniscus repair), MR + PRP, and MR + PRFM groups at 6 or 12 weeks, suggesting limited efficacy of both PRP and PRFM in avascular vertical tears in vivo [44]. In addition, like PRF, it also lacks clinical evidence in human meniscal surgery.

Overall, current data is still variable for PRP. It may reduce failure risk in isolated meniscal repairs but offers limited benefit in the setting of concomitant ACL reconstruction, while a large registry analysis shows the opposite results. On the other hand, PRF and PRFM remain investigational with strong biological rationale but no supporting clinical evidence.

### 3.5. Cell Therapy

Preclinical and early clinical evidence suggests that mesenchymal stromal cell (MSC)–based therapies may enhance meniscal healing, particularly in avascular zones where intrinsic reparative capacity is limited. In a rabbit model, Koch et al. demonstrated that bone marrow aspirate concentrate (BMAC) applied during meniscal repair significantly improved histological healing compared with repair alone, supporting the concept that marrow-derived cells can augment fibrochondrocyte proliferation and matrix regeneration [45]. Complementing these findings, Vangsness et al. conducted a randomized, double-blind, controlled clinical study in humans and found that intra-articular injection of allogeneic bone marrow-derived MSCs following partial medial meniscectomy led to increased meniscal volume on MRI in 24% of patients receiving a lower cell dose, along with improvements in VAS pain and Lysholm scores [46].

More advanced regenerative potential was demonstrated in a primate model by Kondo et al., where transplantation of autologous synovial MSC aggregates onto large meniscal defects resulted in significantly larger regenerated meniscal tissue and superior histological quality at 8 and 16 weeks [47]. Notably, cartilage degeneration in the medial femoral condyle was attenuated in the MSC-treated knees, marking the first primate evidence that MSC transplantation can promote both meniscal regeneration and chondroprotection [47]. Early clinical translation of synovial MSC therapy is also emerging: Sekiya et al. reported that patients undergoing meniscal repair supplemented with cultured autologous synovial MSCs exhibited apparent healing on second-look arthroscopy at 52 weeks, with flap and radial tears showing complete or partial healing and significant improvement in arthroscopic tear scores and Lysholm scores [48].

**Figure 3:**
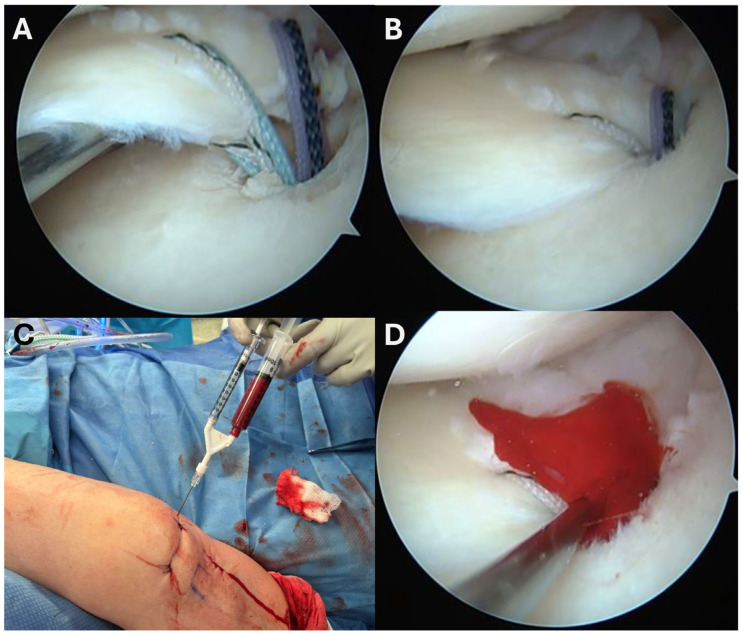
Meniscal root repair augmented with bone marrow aspirate concentrate (BMAC). (**A**) Arthroscopic view after suture passage through the meniscal root and prior to suture tensioning. (**B**) Arthroscopic view following suture tensioning, demonstrating reduction of the meniscal root to its anatomic footprint. (**C**) Injection of BMAC into the repair site to biologically augment healing. (**D**) Final arthroscopic view after completion of the procedure.

Collectively, these studies support the biological rationale that MSCs may enhance meniscal repair by producing anti-inflammatory and immuno-modulatory factors, to modify the harsh intra-articular environment. In addition, MSCs are known to secrete anti-catabolic factors that can counteract the elevated cytokines and degradative enzymes typically present after meniscal injury, thereby improving the biological milieu in which repair must occur (Figure 3).

### 3.6. Collagen Scaffold

**Figure 4:**
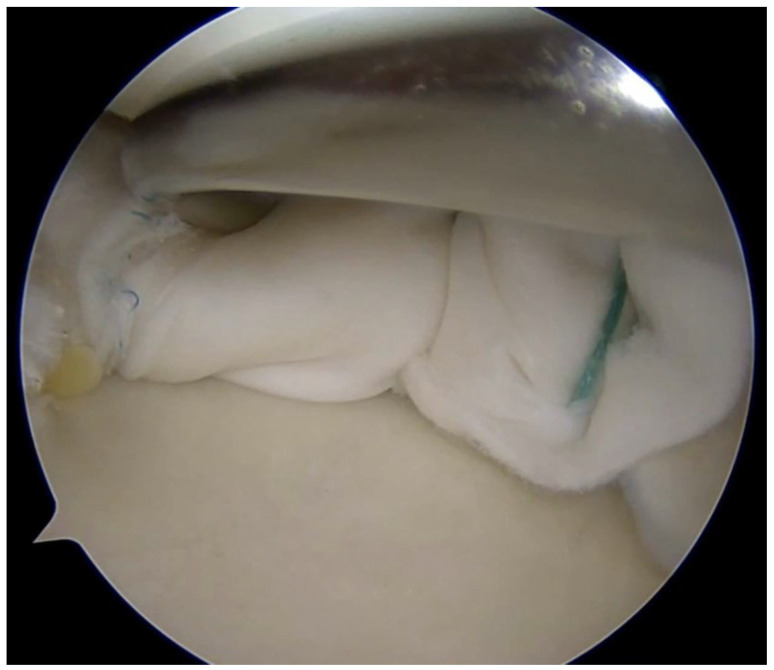
Complex meniscus tears treated with collagen matrix wrapping.

Coverage-based augmentation strategies combining a structural scaffold with biological stimulation have been proposed for the treatment of complex meniscal tears with limited intrinsic healing potential. Piontek et al. reported that Chondroguide collagen membrane—a bilayer, cell-free type I/III collagen scaffold originally developed for osteochondral repair—was “wrapped” around the meniscus tear site, followed by bone marrow injection resulted in significant clinical improvement and apparent structural healing in the majority of patients at 2-year follow-up, although the absence of a control group limits definitive conclusions [49,50]. Significant improvement in IKDC and Lysholm scores at 2-year follow-up were found. Follow-up MRI demonstrated no meniscal tear in 76% of the operated menisci [50]. Earlier work by Henning et al. demonstrated that fascial sheath coverage combined with exogenous fibrin clot injection improved healing rates in complex meniscal tears, supporting the concept that mechanical containment coupled with biologic augmentation can enhance repair [31]. The study reported improved healing rates from 75% to 92% for complex meniscal tears including double flap, double longitudinal, and radial tears when treated with fascia sheath coverage and fibrin clot, compared to conventional repair alone [31]. However, one important limitation is that despite the improved overall healing rate, tears in the middle one-third of the lateral meniscus still showed high failure rates even with this augmentation technique [31]. Together, these studies suggest that providing both structural stability and a biologically favorable microenvironment may expand repair indications for complex meniscal lesions; however, controlled comparative studies are needed to define efficacy and optimal indications (Figure 4).

## 4. Defining the Ideal Biologic Target

Biologic augmentation strategies for meniscal repair aim to overcome the intrinsic limitations of the adult meniscus by targeting multiple complementary healing pathways rather than a single mechanism. Key biologic targets include increased vascularity at the meniscocapsular junction to improve nutrient delivery and facilitate inflammatory cell ingress; stimulation of local cell proliferation to expand the limited population of reparative fibrochondrocytes; and enhanced cell chemotaxis, particularly promoting synovial cell homing to the meniscal defect, which has been shown to be a major source of reparative cells. In parallel, these approaches seek to accelerate extracellular matrix synthesis, restoring collagen and proteoglycan content, while also improving matrix remodeling to achieve structural organization and biomechanical competence comparable to native tissue. Importantly, successful meniscal healing likely requires the coordinated activation of these processes, as strategies that enhance only one biologic target—such as vascular access without cellular recruitment or cell delivery without matrix integration—may be insufficient. Thus, modern biologic augmentation approaches are increasingly designed to modulate the entire repair microenvironment, integrating vascular, cellular, and matrix-level effects to optimize meniscal regeneration.

**Figure 5:**
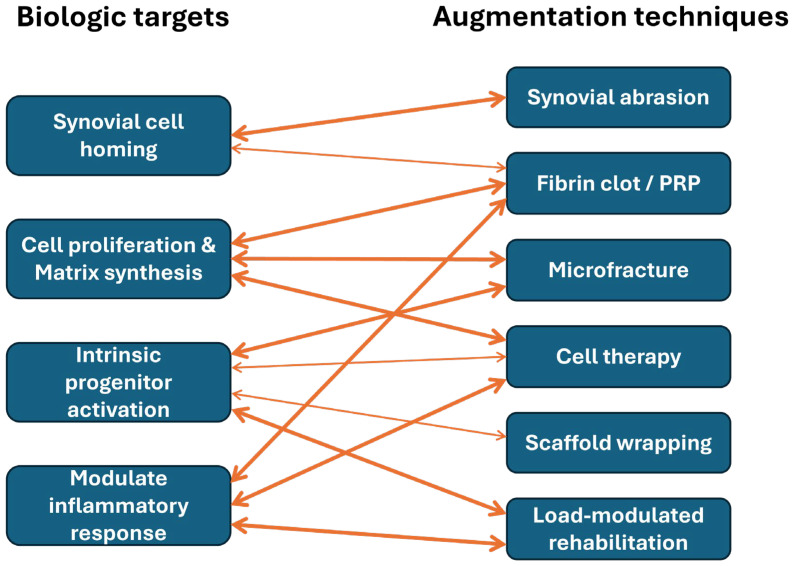
Conceptual mapping between biologic targets and augmentation techniques.

While further studies are required, we believe that stimulation of synovial cell homing to the repair site is a critically important biologic target, as synovium-derived cells represent a major source of reparative progenitors capable of migrating into meniscal defects. In parallel, biologic augmentation should promote local cell proliferation and de novo extracellular matrix synthesis, thereby compensating for the inherently low cellularity and limited anabolic capacity of the adult meniscus. Equally important is the activation of intrinsic progenitor cell niches within the meniscus, which may reside in the peripheral vascular zone or along the meniscocapsular interface and could be harnessed to support endogenous repair. Finally, successful healing requires amelioration of the excessive inflammatory response and associated protease activity that characterize the post-injury intra-articular environment, as unchecked inflammation and matrix-degrading enzymes can overwhelm reparative processes. Collectively, these biologic targets emphasize that meniscal healing is not governed by a single pathway, but rather by the coordinated regulation of cellular recruitment, anabolic activity, progenitor cell engagement, and inflammatory balance—providing a conceptual framework for the rational design of next-generation biologic augmentation strategies (Figure 5).

## 5. Our Current Approach: What Can We Practically Use?

Our current approach to biologically augmented meniscal repair is intentionally designed to address specific biologic targets known to limit intrinsic healing. Synovial abrasion is performed to stimulate synovial cell homing to the repair site, leveraging the synovium as a primary source of reparative cells and vascular access. Microfracture of the intercondylar notch is used to introduce marrow-derived cells and trophic factors, thereby stimulating cell proliferation, matrix synthesis, and possible activation of intrinsic progenitor niches within the meniscus and surrounding tissues. When biologic supplementation is indicated, leukocyte-rich platelet-rich plasma (LR-PRP) is selectively used for its ability to promote early inflammatory signaling, enhance cell recruitment, and support new extracellular matrix deposition, while also modulating the local inflammatory milieu. To improve biologic retention and spatial localization, PRP is combined with thrombin and calcium chloride to form a fibrin clot, which functions as a provisional scaffold that facilitates cell adhesion, sustained growth factor release, and early matrix organization. Finally, postoperative rehabilitation is tailored to the biomechanical demands of the tear pattern, recognizing that controlled mechanical loading is a critical regulator of matrix remodeling and inflammatory resolution. Vertical longitudinal tears are permitted partial compressive loading in extension to support physiological remodeling, whereas radial and complex tears are protected with minimal early loading to avoid disruption of nascent repair tissue. Together, this integrated strategy aligns biologic augmentation with mechanical modulation to optimize the cellular, molecular, and structural determinants of meniscal healing (Table 1).

**Table 1:** Ideal biologic targets and their corresponding technique, mechanism, and rationale.

Biologic Target	Surgical/Biologic Technique	Primary Mechanism	Supporting Rationale
Stimulate synovial cell homing to repair site	Synovial abrasion/rasping	Induces local bleeding and inflammatory signaling at the meniscocapsular junction	Synovium is a major source of reparative cells; abrasion enhances migration of synovial-derived cells into the meniscal defect
	Fibrin clot (PRP + thrombin + CaCl_2_)	Chemotactic scaffold	Provides a provisional matrix facilitating cell adhesion and directional migration
Stimulate cell proliferation and new matrix synthesis	Intercondylar notch microfracture (marrow venting)	Release of marrow-derived cells and growth factors	Mimics biologic environment of ACL reconstruction–associated hemarthrosis
	Leukocyte-rich PRP	Growth factor–mediated anabolic signaling	PDGF, TGF-β promote cell proliferation and extracellular matrix production
Stimulate intrinsic progenitor cell niche in the meniscus	Marrow stimulation (microfracture/venting)	Activation of endogenous progenitor populations	Marrow elements may activate peripheral meniscal progenitors and synoviomeniscal junction niches
	Controlled mechanical loading	Mechanotransduction	Physiologic loading may stimulate progenitor cell activation and matrix organization
Ameliorate excessive inflammatory response and protease activity	Spatially confined PRP (clot formation)	Localized delivery and temporal regulation of inflammation	Avoids diffuse intra-articular exposure and excessive catabolic signaling
	Load-modulated rehabilitation	Reduction of injurious shear and hoop stress	Prevents overload-induced inflammation and protease-mediated matrix breakdown

## 6. Future Directions

Emerging therapeutic strategies for meniscus repair and regeneration show promise in preclinical studies, but most lack high-quality clinical evidence and remain investigational. These approaches target key biological barriers to healing including limited vascularity, inflammatory milieu, matrix degradation, and insufficient cellular response.

### 6.1. Simvastatin: Stimulation of Fibrocartilage Formation

Local administration of simvastatin significantly enhances avascular meniscus healing in animal models by upregulating bone morphogenetic proteins (BMPs) and subsequent collagen production. Zhang et al. demonstrated in a rabbit model that simvastatin-conjugated gelatin hydrogel implanted into avascular meniscal defects resulted in significantly higher histological scores, occupation ratios, and biomechanical stiffness compared to controls at 12 weeks [51]. The mechanism involves upregulation of BMP-2 and BMP-7 (at 4–8 h and 7–14 days), followed by increased type I and II collagen expression (at 7–14 days) [51]. Immunohistochemistry showed stronger staining for COL1, COL2, BMP-2, and BMP-7 in simvastatin-treated tissue at 12 weeks [51]. This approach is considered safe, inexpensive, and promising for clinical translation [51,52].

### 6.2. Tranexamic Acid: Fibrinolysis Inhibitor

While tranexamic acid is widely used in orthopedic surgery to reduce blood loss, specific evidence for its use as a fibrinolysis inhibitor to enhance meniscal repair is limited in the available literature. The rationale would be to preserve fibrin clot scaffolds that support cellular migration and healing, but dedicated studies evaluating this application for meniscus repair have not been extensively published [52]. In a rabbit model evaluating the role of antifibrinolytic modulation on meniscal repair, intra-articular administration of tranexamic acid (TXA) did not enhance healing of either circular meniscal defects or repaired longitudinal tears at 2, 4, or 8 weeks postoperatively [53]. Despite its theoretical advantage in stabilizing fibrin clot formation, TXA failed to improve histologic or biomechanical markers of meniscal repair. Moreover, TXA-treated knees demonstrated adverse effects on articular cartilage, including early deterioration of tibial cartilage structure and reduced proteoglycan content in femoral cartilage at later time points [53]. These findings suggest that while fibrin stability is important for early repair, prolonged intra-articular blood retention or impaired fibrinolysis may create a deleterious joint environment, negatively affecting cartilage health without conferring benefit to meniscal healing. Collectively, this study cautions against routine intra-articular TXA use as a biologic augmentation strategy for meniscal repair and highlights the need for further investigation into timing- and dose-dependent effects of antifibrinolytic agents [53].

### 6.3. Losartan: TGF-β Inhibition

Modulation of transforming growth factor–β (TGF-β) signaling represents a theoretically attractive strategy to promote more physiologic meniscal healing while limiting pathologic fibrosis, although direct clinical evidence remains lacking. TGF-β plays a critical role in meniscal matrix synthesis and has been widely investigated for its pro-chondrogenic and anabolic effects. However, excessive or prolonged TGF-β activation may preferentially drive fibrotic tissue deposition rather than organized fibrocartilage regeneration, potentially compromising tissue architecture and mechanical function [54,55]. Pharmacologic agents such as losartan, an angiotensin II receptor blocker with well-described anti-fibrotic effects mediated through inhibition of TGF-β signaling, have shown promise in improving topical wound healing in a rat model [56]. To date, however, losartan has not been systematically evaluated in either preclinical models or clinical studies of meniscal repair. Further investigation is therefore warranted to determine whether controlled modulation of TGF-β signaling can shift the reparative response toward functional fibrocartilage regeneration without inducing fibrosis.

### 6.4. Low-Intensity Pulsed Ultrasound (LIPUS)

Low-intensity pulsed ultrasound (LIPUS) has emerged as a noninvasive modality with potential reparative effects on meniscal tissue through modulation of cellular differentiation and matrix synthesis. Kamatsuki et al. demonstrated that LIPUS stimulation induces CCN2/CTGF (Cellular communication network factor-2/Connective tissue growth factor) expression and upregulates meniscus-related and chondrogenic genes in cultured human meniscus cells and meniscal tissue explants, with region-specific responses between the inner and outer meniscus [57]. Notably, cells from the inner meniscus—sharing phenotypic similarities with articular cartilage—exhibited distinct differentiation and gene expression patterns compared with those from the peripheral region, suggesting that LIPUS may differentially regulate meniscal biology according to intrinsic zonal characteristics. Consistent with these mechanistic findings, experimental studies have reported a possible reparative effect of LIPUS on injured meniscus, including enhanced matrix synthesis and improved histological features following meniscal injury [58]. Together, these data support a biologically plausible role for LIPUS in promoting meniscal repair through CCN-mediated anabolic signaling and chondroprotective pathways. However, despite encouraging in vitro and preclinical evidence, the clinical translation of LIPUS for meniscal healing remains investigational, and its therapeutic efficacy, optimal treatment parameters, and indications require validation in well-designed clinical studies.

### 6.5. Tissue Adhesives

Novel tissue adhesives (TAs) are being developed as an alternative or adjunct to suture-based meniscal repair, with the goal of providing immediate mechanical integrity while simultaneously supporting biological healing. These materials are designed to address the inherent mechanical challenges of conventional suturing, particularly in complex tear patterns and avascular regions where fixation is difficult and healing capacity is limited [59]. In contrast to prefabricated scaffolds, which typically require a mini-open incision for implantation, injectable or in situ–crosslinkable TAs can be delivered arthroscopically and polymerized directly within the joint, making them especially attractive for tear locations that are not readily accessible for suture repair [59]. In addition to mechanical stabilization, TAs may be used alone or as carriers for biologic agents, thereby supporting matrix deposition, tear integration, and localized biologic modulation [60]. A recent review reveals an absence of comparative high-quality evidence supporting the routine use of TAs for meniscal repair and emphasizes the lack of an ideal TA designed for that purpose. Further high-quality research, basic science and clinical studies will facilitate the development of new materials and enable testing their suitability for use in meniscal repair [61].

### 6.6. Modulation of the Intra-Articular Milieu

Targeting inflammatory and catabolic mediators in the joint represents a rational approach to enhance meniscal healing, with preclinical evidence supporting IL-1β and TNF-α inhibition.

IL-1β Inhibition (Anakinra): Anakinra is FDA-approved for rheumatoid arthritis, neonatal-onset multisystem inflammatory disease (NOMID), and deficiency of interleukin-1 receptor antagonist (DIRA). While not specifically approved for meniscal repair, acute intra-articular IL-1 inhibition after knee injury has been shown to reduce cartilage degeneration and synovial inflammation in animal models [62,63]. Given that IL-1β is elevated after meniscal injury and drives catabolic processes including matrix degradation and cell death, its inhibition could theoretically improve healing outcomes [62,63].

TNF-α Inhibition (Etanercept): Similar to IL-1β, TNF-α is a pro-inflammatory cytokine elevated in injured joints that promotes catabolism and inhibits anabolic repair pathways [62,63]. While etanercept (Enbrel) is used clinically for inflammatory arthritis, specific evidence for its use in meniscal repair augmentation is lacking [52].

MMP Inhibition: Matrix metalloproteinases are markedly elevated in synovial fluid after meniscal injury (25-fold increase in MMP activity), and correlate strongly with inflammatory mediators like PGE2. Modulation of MMP activity represents a potential therapeutic target, though no specific MMP inhibitors have been clinically evaluated for meniscal repair [62,63]. One potential agent for future evaluation would include alpha-2 macroglobulin, which is a broad-spectrum MMP inhibitor.

### 6.7. Immune Cell Subpopulations

It is well recognized that the immune system plays a central role in tissue healing by orchestrating inflammatory responses, tissue remodeling, and regenerative processes. The role of mononuclear leukocytes in mediating meniscal catabolism has been established, with these cells inducing MMP activity, nitric oxide production, and glycosaminoglycan loss [22]. In addition, modulation of specific immune cell populations—including macrophages, regulatory T cells, dendritic cells, and natural killer cells—represents an emerging frontier in meniscal repair, though evidence remains primarily theoretical. Some of them contribute to tissue healing by modulating immune responses and influencing fibroblast activity, and guiding angiogenesis [64]. However, specific strategies targeting these cell populations for meniscal healing have not been extensively studied.

### 6.8. Stimulation of Intrinsic Stem Cell Niche in Meniscus and Synovium

Recent advances in developmental biology have identified distinct progenitor cell populations within the meniscus and synovium that may be therapeutically harnessed to improve tissue repair. The synovium represents a particularly rich reservoir of mesenchymal stromal cells with robust proliferative capacity and chondrogenic potential, and transplantation of synovial MSCs has demonstrated encouraging outcomes in clinical case series [48]. Elucidating the molecular mechanisms that govern these endogenous stem cell niches—such as growth factor signaling, biomechanical stimuli, and niche-specific microenvironmental cues—may facilitate strategies aimed at activating intrinsic repair pathways without the need for exogenous cell transplantation.

## 7. Conclusions

Meniscal healing is governed by a complex interplay of vascular supply, cellular availability, extracellular matrix architecture, biomechanical loading, and intra-articular inflammatory and immune regulation. Although numerous biologic augmentation strategies have demonstrated promise in preclinical models and selected clinical settings, high-quality clinical evidence remains limited, and no single intervention has emerged as universally effective. Importantly, the absence of definitive data defining optimal mechanical loading parameters during healing represents a critical unmet need, despite increasing recognition of the close interaction between biomechanical forces and meniscal cell biology. Collectively, current evidence suggests that successful meniscal repair will require multifaceted, mechanism-driven approaches that integrate structural stabilization with targeted modulation of the biological microenvironment. Further investigation into the cellular and molecular mechanisms of meniscal healing—particularly the interaction between biomechanical load and meniscal cell biology—will be essential for establishing a rationale “gold standard” for treatment and rehabilitation and for improving long-term clinical outcomes.

## Data Availability

All data used in this review are derived from previously published studies and are cited in the reference list.
